# Intratumoral CD8^+^ Cytotoxic Lymphocyte Is a Favorable Prognostic Marker in Node-Negative Breast Cancer

**DOI:** 10.1371/journal.pone.0095475

**Published:** 2014-04-17

**Authors:** Zonglin Chen, Xianyu Chen, Enxiang Zhou, Ganlong Chen, Ke Qian, Xia Wu, Xiongying Miao, Zhonghua Tang

**Affiliations:** 1 Department of General Surgery, the Second Xiangya Hospital, Central South University, Changsha, China; 2 Department of Pathology, the Second Xiangya Hospital, Central South University, Changsha, China; Texas Tech University Health Sciences Center, United States of America

## Abstract

**Background:**

The prognostic effect of tumor infiltrating CD8^+^ cytotoxic lymphocytes (CTLs) in breast cancer is controversial. We analyzed the association between CD8^+^ CTLs and survival of untreated node-negative breast cancer patients.

**Material and Methods:**

CD8^+^ CTLs infiltrate was evaluated by immunostaining in a cohort of 332 node-negative breast cancer patients with a median follow-up of 152 months. The prognostic significance of CD8^+^ CTLs for disease-free survival (DFS) and breast cancer-specific overall survival (OS) was evaluated with Kaplan-Meier survival analysis as well as univariate analysis and multivariate Cox analysis adjusted for age at diagnosis, pT stage, histological grade, estrogen receptor (ER) status, progesterone receptor (PR) status, Ki-67 expression and human epidermal growth factor receptor 2 (HER-2) status.

**Results:**

285 (85.8%) patients showed strong CD8^+^ CTLs infiltrate positive status. Univariate analysis showed that CD8^+^ CTLs had statistically significant association with DFS (P = 0.004, hazard ratio [HR] = 0.454, 95% confidence interval [CI] = 0.265–0.777) and OS (P = 0.014, HR = 0.430, 95% CI = 0.220–0.840) in the entire cohort. The significance of CD8^+^ CTLs was especially strong in ER negative, HER-2 negative and ER, PR, HER-2 triple-negative breast cancers. In Kaplan-Meier analysis, CD8^+^ CTLs had significant effect on prognosis of patients (Log-rank test: P = 0.003 for DFS and P = 0.011 for OS), independent of established clinical factors for DFS (P = 0.002, HR = 0.418, 95% CI = 0.242–0.724) as well as for OS (P = 0.009, HR = 0.401, 95% CI = 0.202–0.797).

## Introduction

Breast cancer is the most frequent and fatal female cancer worldwide. Though its prognosis has been improved by early diagnosis and multiple therapies, the approaches to evaluate prognosis are still limited. New prognosis factors are needed to reach a better evaluation and help select patients who likely benefit from highly targeted therapies. Traditional clinicopathological variables such as age, pT stage and histological grade have long been used for predicting the survival or as a guide to diagnosis and therapy [1]. By applying gene expression testing, recently some immune cell-relative gene signatures were also found to be good prognostic and predictive factors [2]–[4].

The relationship between human cancer and immune system is complex and not fully understood. On the one hand, some types of immune cells such as nature killer (NK) cells, B cells were shown to suppress growth of cancer cells and higher number of these cells associated with better prognosis [5]–[7]; on the other hand, studies demonstrated that other types of immune cells including macrophages, FOXP3^+^ regulatory T cells (Tregs) actually facilitated and promoted the expansion and development of cancer [8]–[10]. These seemingly conflicting findings resulted in the formation of the cancer immunoediting hypothesis suggesting that immune system had both anti-tumour function and tumour-promoting action on the progression of human cancer [11]–[14].

In the adaptive immune response against human cancer, CD8^+^ cytotoxic T lymphocytes (CTLs) have played one of the most important roles that attacking cancer cells by producing interferon gamma to induce apoptosis of tumor cells and macrophage tumor killing activity [14]. Accordingly, CD8^+^ CTLs have been shown to associate with better survival of colorectal [15], [16], lung [17], [18], oesophageal [19], [20], epithelial ovarian [21], [22], renal cell [23] and pancreatic cancers [24]. In breast cancer, however, the prognostic effect of CD8^+^ CTLs is still a matter of debate. While one study demonstrated that both the total number and the distant stromal (more than one tumor cell diameter of the tumor) CD8^+^ CTLs significantly associated with better prognosis of breast cancer and its subtypes (ER negative cancer, HER-2 negative cancer and basal-like cancer) [25], another study showed that neither intratumoral (within tumor cell nests) nor peritumoral (stroma without direct contact with the cancer cells) CD8^+^ CTLs had protective effect on survival of breast cancer patients [26]. Furthermore, one study demonstrated that the favourable effect of CD8^+^ CTLs was only limited to ER negative, high histological grade breast cancers [27], whereas another recent publication reported that intratumoral CD8^+^ CTLs only had statistically significant and independent association with better prognosis in triple-negative breast cancer (ER negative, progesterone receptor [PR] negative, HER-2 negative), especially in core basal phenotype breast cancer [ER negative, PR negative, HER-2 negative, epidermal growth factor receptor positive (EGFR) or cytokeratin (CK) 5/6 positive] [28]. Because of these conflicting results, the aim of our current study was to analyze the effect of immunohistochemically detected intratumoral and peritumoral (within tumor stroma) CD8^+^ CTLs for disease-free survival (DFS) and breast cancer-specific overall survival (OS) in 396 untreated node-negative breast cancer patients who did not receive systemic therapy in the adjuvant setting with long follow-up. We also analyzed the prognostic effect of CD8^+^ CTLs in subgroups according to ER and HER-2 expression as well as in triple negative breast cancer.

## Patients and Methods

### Study Patients

Our initial study cohort included 396 consecutive lymph node-negative breast cancer patients not treated in the adjuvant setting. The tumor size was pT1 to pT3 and there was adequate follow-up information of patients who were treated at the General Surgery Department of the Second Xiangya Hospital of Central South University between the year 1980 and 2001. Of these 396 patients, paraffin blocks with tumor tissue for CD8^+^ CTLs immunohistochemistry (IHC) were available of 332 individuals who were analyzed in this study. All these patients were treated by surgical tumor resection and did not receive any systemic adjuvant therapy.

Among 396 breast cancer patients, 215 (54.3%) patients were treated with breast conserving surgery followed by irradiation and 181 (45.7%) with modified radical mastectomy. We only focused on node-negative breast cancer patients with pT1–3 tumors without any evidence of metastatic disease at the time of surgery. The median age at diagnosis of the patients was 60 years (range 33 to 91 years). Follow up was done by writing letters to patients, phoning and by checking records of patients at least once a year from 1980 to 2001. In this period, we documented death from cancer or from other reasons unrelated to breast cancer and recurrence of disease, which include metastasis, local relapse and secondary tumors. The mean follow-up time was 152 months. 53 (15.6%) patients died from breast cancer, 46 (13.6%) patients died from other diseases unrelated to breast cancer, 7 (2.1%) patients died from unknown causes, 233 (68.7%) patients were alive and 90 (26.5%) patients had recurrence. The patients dying from other reasons were censored from their survival statistics analysis at their date of death. The study was approved by the ethical review board of the medical association of the Second Xiangya Hospital of Central South University. The manuscript was prepared in agreement with the reporting recommendations for tumor marker reporting studies [29].

### Ethics Statement

The study was approved by the ethical review board of the medical association of the Second Xiangya Hospital of Central South University. Informed consent has been obtained and all clinical investigation has been conducted according to the principles expressed in the Declaration of Helsinki.

### Immunohistochemistry for CD8^+^ CTLs

Immunostaining was done on 4 µm thick sections according to standard procedures as previously described [26]. Serial sections of formalin-fixed and paraffin-embedded tumor tissue were subsequently deparaffinized using graded alcohol and xylene. Antigen retrieval reactions were performed in a steamer in citrate buffer of pH10 for 25 minutes. 3% H2O2 solution was applied to block endogenous peroxides at room temperature for 5 minutes. Monoclonal CD8 antibodies (Clone CD8/144B, DakoCytomation, Glostrup, Denmark) in a dilution 1∶50 was used to incubate with the tissue sections for 60 minutes at room temperature in a humidified chamber, followed by polymeric biotin–free visualization system (Envision™, DAKO Diagnostic Company, Hamburg, Germany) reaction for 30 minutes at room temperature. Then the sections were reacted with 3, 3-diaminobenzidine (DAB) (Envision™, DAKO Diagnostic Company, Hamburg, Germany) in a dilution 1∶50 with substrate buffer for 5 minutes at room temperature and counterstained with Mayer’s haematoxylin solution for 5 minutes. All slides were mounted and then were observed and evaluated under a Leica light microscope (Leica Microsystem Vertrieb Company, Wetzler, Germany) by two of the authors trained in histological and immunohistochemical diagnostics, unaware of the clinical outcome. All series included appropriate positive (tonsil) and negative (hepatocytes) controls and all controls gave adequate results.

### Evaluation of Immunostaining

CD8^+^ CTLs showed membrane staining and were evaluated in two locations in each tumour: intratumoral and peritumoral compartments as previously described [25]–[28]. In brief, intratumoral CD8^+^ CTLs were defined as CD8^+^ CTLs located within tumor cell nets or in direct contact with the breast cancer malignant epithelial cells, whereas peritumoral CD8^+^ CTLs were defined as CD8^+^ CTLs in the stroma without direct contact with the cancer cells. A semi-quantitative scoring method similar to that used by other studies [30]–[32] was employed to evaluate the intensity of CD8 positive infiltrate: 0, no CD8^+^ CTLs positive infiltrate; 1+, weak CD8^+^ CTLs positive infiltrate; 2+, moderate CD8^+^ CTLs positive infiltrate; 3+, strong CD8^+^ CTLs positive infiltrate. In case of disagreement of the results of two independent examiners the slides were re-examined and discussed at the microscope until a consensus was reached.

### Immunohistochemistry and Evaluation for ER, PR, Ki-67, HER-2

Additional immunohistochemistry for ER, PR, Ki-67 and HER-2 was also conducted using the standard procedures. Briefly, serial sections of formalin-fixed and paraffin-embedded tumor tissues were stained with monoclonal ER antibodies (clone 1D5, 1∶150 dilution, Dako, Glostrup, Denmark), monoclonal PR antibodies (clone PgR 636, 1∶150 dilution, Dako, Glostrup, Denmark), monoclone Ki-67 antibodies (clone MIB-1, 1∶200 dilution, Dako, Glostrup, Denmark) as well as polyclonal HER-2 antibodies (A0485, Dako, Glostrup, Denmark) according to manufacturer’s instructions. ER and PR expression was analyzed as percentage of all tumor cells and any nuclear expression >0 was considered positive. Ki-67 expression of more than 20% was considered as high expression and a percentage ≤20% was defined as low expression. HER-2 was scored from 0 to 3+ according to the well-published manufacturer’s instructions. HER-2 3+ tumors were considered HER-2 positive. All HER-2 2+ cases were confirmed by Fluorescence in-situ hybridization (FISH) using a dual-color probe (DakoCy-tomation) containing a spectrum green-labeled HER-2 gene (17q11.2-q12) probe and a spectrum green-labeled centromere control for chromosome 17 (17p11.1-q11.1). HER-2 tumors with 2+ HER-2 amplification were finally considered HER-2 positive.

### Statistical Analysis

Survival rates were calculated according to the Kaplan-Meier method. Breast cancer-specific DFS was calculated from the diagnosis date to the date of recurrence including local relapse, distant metastasis, and detection of the contra lateral breast cancer. Breast cancer OS was computed from the date of diagnosis to the date of death from breast cancer. Survival was compared with the Log-rank test. Univariate analysis and multivariate Cox analysis with proportional hazard regression model were employed to assess the effects of CD8^+^ CTLs and other prognostic factors. Multivariate Cox survival analysis was done with inclusion. Dichotomization was done as follows: age at diagnosis in <50 years and ≥50 years, pT stage in pT1 (≤2cm) versus pT2 and pT3 (>2cm), histological grade in G I and G II versus G III, ER status in negative and positive, PR status in negative and positive, HER-2 status in negative and positive, and Ki-67 expression in low and high. CD8^+^ CTLs in the whole cohort as well as in ER negative, ER positive, HER-2 negative, HER-2 positive, triple-negative (ER negative, PR negative, and HER-2 negative) was assessed and Kaplan-Meier calculation, univariate analysis and multivariate Cox analysis of CD8^+^ CTLs for DFS and OS were done. Correlations between CD8^+^ CTLs, age at diagnosis, pT stage, histological grade, ER status, PR as well as HER-2 status and Ki-67 were analyzed using Chi-Squared test. Since no correction for multiple testing was done, all results were interpreted explorative. All P values were two sides and a P<0.05 was considered statistically significant. All statistical analyses were done using the Statistical Package for the Social Science (SPSS) (SPSS Inc, version 15.0, Chicago, IL, USA).

## Results

### Results of Immunohistochemistry and Cut-off Establishment of CD8^+^ CTLs Positive Infiltrate Scores

**Figure 1:**
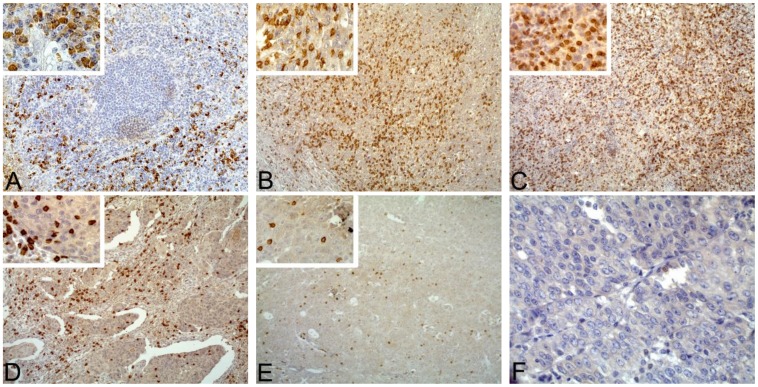
Representative examples of CD8^+^ CTLs immunostaining in a positive control and breast cancer. A: Normal human tonsil tissue, strong CD8^+^ CTLs positive infiltrate was mainly distributed in the parafollicular area (original magnification: 100-fold; inset: 400-fold). B: Strong CD8^+^ CTLs positive infiltrate in invasive breast cancer (CD8^+^ CTLs positive infiltrate score: 3+) (original magnification: 100-fold; inset: 400-fold). C: Strong CD8^+^ CTLs positive infiltrate in medullary breast cancer (CD8^+^ CTLs positive infiltrate score: 3+) (original magnification: 100-fold; inset: 400-fold). D: Moderate CD8^+^ CTLs positive infiltrate (CD8^+^ CTLs infiltrate score: 2+) (original magnification: 100-fold; inset: 400-fold). E: Weak CD8^+^ CTLs positive infiltrate (CD8^+^ CTLs positive infiltrate score: 1+) (original magnification: 100-fold; inset: 400-fold). F: CD8^+^ CTLs negative breast cancer (original magnification: 400-fold).

Established clinicopathological variables were assessed, including age at diagnosis, pT stage, histological grade, ER status, PR status as well as HER-2 status and Ki-67 expression (Table 1). CD8^+^ CTLs were determined by immunohistochemistry (IHC). Representative examples of CD8^+^ CTLs and positive control human tonsil tissue immunostaining were showed in Figure 1. CD8^+^ CTLs presented in a diffuse pattern and those infiltrating within peritumoral compartment were more abundant than those infiltrating within intratumoral compartment. In the positive control tonsil, CD8^+^ CTLs were distributed mainly in the paracortical area, with small numbers within germinal centers (Fig. 1A). Using CD8^+^ CTLs positive infiltrate scoring method, 47 (14.2%) patients were graded 0, 143 (43.1%) patients were graded 1+, 75 (22.6%) patients were graded 2+ and 67 (20.2%) patients were graded 3+ in intratumoral compartment (Table 1); in peritumoral compartment, 16 (4.8%) patients were graded 0, 126 (38.0%) were graded 1+, 117 (35.2%) were graded 2+ and 73 (22.0%) were graded 3+ (Table 1).

**Table 1:** Clinicopathological variables of all patients (n = 332).

Clinicopathological variables	Number	%
Age at diagnosis		
<50	86	25.9
≥50	246	74.1
pT stage		
pT_1_	208	62.7
pT_2_	120	36.1
pT_3_	4	1.2
Histological grade		
G I	76	22.9
G II	185	55.7
G III	71	21.4
Estrogen receptor status		
Negative	79	23.8
Positive	253	76.2
Progesterone receptor status		
Negative	103	31.0
Positive	229	69.0
HER-2 status		
Negative	291	87.7
Positive	41	12.3
Ki-67 expression		
Low	225	67.8
High	95	28.6
Missing	12	3.6
Intratumoral CD8^+^ CTLs positive infiltrate score		
0	47	14.2
1+	143	43.1
2+	75	22.6
3+	67	20.2
Peritumoral CD8^+^ CTLs positive infiltrate score		
0	16	4.8
1+	126	38.0
2+	117	35.2
3+	73	22.0
CD8^+^ CTLs infiltrate status		
Negative	47	14.2
Positive	285	85.8
Death		
Due to cancer	53	16.0
Unrelated to cancer	45	13.6
	41	12.3
Unknown causes	7	2.1
Surviving	227	68.4
Relapse		
Yes	90	27.1
No	242	72.9

HER-2 human epidermal growth factor receptor 2; CTLs, cytotoxic T lymphocytes.

CD8^+^ CTLs infiltrate status was done based on dichotomising of intratumoral CD8^+^ CTLs positive infiltrate scores.

Log-Rank tests and Kaplan Meier estimates were performed for survival differences between pairs of intratumoral and peritumoral CD8^+^ CTLs positive infiltrate scores: intratumoral CD8^+^ CTLs positive infiltrate score 0 vs. 1+ (Log-rank test: P = 0.013 for DFS, Figure S1A; P = 0.033 for OS, Figure S1B), 0 vs. 2+ (Log-rank test: P = 0.006 for DFS, Figure S1C; P = 0.056 for OS, Figure S1D), 0 vs. 3+ (Log-rank test: P = 0.019 for DFS, Figure S1E; P = 0.012 for OS, Figure S1F), 1+ vs. 2+ (Log-rank test: P = 0.161 for DFS, Figure S2A; P = 0.727 for OS, Figure S2B), 1+ vs. 3+ (Log-rank test: P = 0.814 for DFS, Figure S2C; P = 0.421 for OS, Figure S2D), 2+ vs. 3+ (Log-rank test: P = 0.388 for DFS, Figure S2E; P = 0.547 for OS, Figure S2F); peritumoral CD8+ CTLs positive infiltrate score 0 vs. 1+ (Log-rank test: P = 0.104 for DFS, Figure S3A; P = 0.227 for OS, Figure S3B), 0 vs. 2+ (Log-rank test: P = 0.070 for DFS, Figure S3C; P = 0.186 for OS, Figure S3D), 0 vs. 3+ (Log-rank test: P = 0.140 for DFS, Figure S3E; P = 0.084 for OS, Figure S3F), 1+ vs. 2+ (Log-rank test: P = 0.905 for DFS, Figure S4A; P = 0.950 for OS, Figure S4B), 1+ vs. 3+ (Log-rank test: P = 0.811 for DFS, Figure S4C; P = 0.355 for OS, Figure S4D), 2+ vs. 3+ (Log-rank test: P = 0.885 for DFS, Figure S4E; P = 0.423 for OS, Figure S4F). Furthermore, Kaplan Meier calculation and Log-Rank tests were also used to analyze the prognostic significance of every intratumoral and peritumoral CD8^+^ CTLs positive infiltrate score (0, 1+, 2+, and 3+). There were significantly different DFS and a trend OS difference for patients with intratumoral CD8^+^ CTLs positive infiltrate score 0, 1+, 2+, 3+ (Log-rank test: P = 0.014 for DFS, Figure S5A; and P = 0.070 for OS, Figure S5B). Among intratumoral CD8^+^ CTLs positive infiltrate score 1+, 2+, 3+, in contrast, no significant differences in DFS and OS were found (Log-rank test: P = 0.358 for DFS, Figure S5C; P = 0.709 for OS, Figure S5D). Similarly, no significantly prognostic differences were found among peritumoral CD8^+^ CTLs positive infiltrate score 0, 1+, 2+, 3+ (Log-rank test: P = 0.368 for DFS, Figure S6A; P = 0.392 for OS, Figure S6B) and 1+, 2+, 3+ (Log-rank test: P = 0.967 for DFS, Figure S6C; P = 0.638 for OS, Figure S6D). All above results pointed to that only intratumoral CD8^+^ CTLs had significantly protective effect on prognosis of patients and were therefore used and dichotomized for further statistical analysis: cases with intratumoral CD8^+^ CTLs positive infiltrate score 1+, 2+, 3+ were considered as positive CD8^+^ CTLs infiltrate status (n = 285) and cases with score 0 as negative CD8^+^ CTLs infiltrate status (n = 47) (Table 1).

### CD8^+^ CTLs has Protective Effect on Survival in the Entire Cohort

In the whole patient series, patients with positive and negative CD8^+^ CTLs infiltrate status represented 85.8% (n = 285) and 14.2% (n = 47) of all 332 cases, respectively. In patients with positive CD8^+^ CTL infiltrate status, 25.6% (n = 73) patients had a recurrence and 14.7% (n = 42) died. In patients with negative CD8^+^ CTLs infiltrate status, however, 36.2% (n = 17) had a recurrence and 23.4% (n = 11) was died.

**Figure 2:**
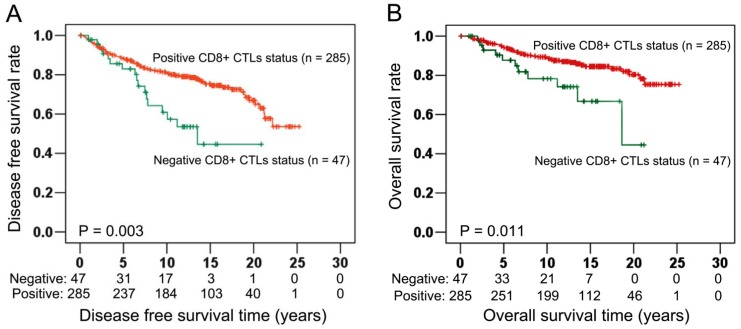
Correlation of CD8^+^ CTLs with prognosis in the entire cohort (n = 332). Using the established CD8^+^ CTLs infiltrate status for Kaplan Meier survival analysis, results demonstrated that positive CD8^+^ CTLs infiltrate status were statistically significant associated with longer DFS (Log-rank test: P = 0.003; Fig. 2A) and longer OS (Log-rank test: P = 0.011; Fig. 2B).

Patients with positive CD8^+^ CTLs infiltrate status had a median duration of DFS of 12.19 years, as compared with only 7.79 years among patients with negative CD8^+^ CTLs infiltrate status. The five and ten-year DFS rate were 83.2% and 64.6% among 285 patients with positive CD8^+^ CTLs infiltrate status but only 66.0% and 36.2% among 47 patients with negative CD8^+^ CTLs infiltrate status. Performing univariate analysis, patients with positive CD8^+^ CTLs infiltrate status had a significant and longer DFS than those patients with negative CD8^+^ CTLs infiltrate status with P = 0.004, HR = 0.454 and 95% CI = 0.265–0.777 (Table 2A). In Kaplan Meier survival analysis, positive CD8^+^ CTLs infiltrate status also showed strongly protective effect on DFS (Log-rank test: P = 0.003, Fig. 2A). In the multivariate Cox regression model including well-recognized prognostic variables related to patient survival such as age at diagnosis, pT stage, histological grade, ER status, PR status and HER-2 status, positive CD8^+^ CTLs infiltrate status independently associated with improved DFS with P = 0.002, HR = 0.414 and 95% CI = 0.239–0.717 (Table 2B). Besides CD8^+^ CTLs, only histological grade had independent prognosis significance (P<0.001, HR = 2.254, 95% CI = 1.563–3.250; Table 2B).

**Table 2:** Univariate analysis and multivariate Cox analysis of CD8^+^ CTLs infiltrate status for disease-free survival (DFS) in the entire cohort (n = 332).

Clinicopathological variables	HR	95%-CI	P
A. Univariate analysis			
CD8^+^ CTLs infiltrate status (− vs. +)	0.454	0.265–0.777	0.004
Age (<50 years vs. ≥50 years)	0.987	0.627–1.553	0.954
pT stage (≤2 cm vs. >2 cm)	1.297	0.855–1.969	0.221
Histological grade (G I and II vs. G III)	2.972	1.946–4.538	<0.001
ER status (− vs. +)	0.677	0.436–1.054	0.084
PR status (− vs. +)	0.786	0.512–1.207	0.271
HER-2 status (− vs. +)	1.547	0.900–2.657	0.114
Ki-67 expression^a^ (Low vs. High)	1.931	1.261–2.957	0.002
B. Multivariate Cox analysis			
CD8^+^ CTLs infiltrate status (− vs. +)	0.418	0.242–0.724	0.002
Age (<50 years vs. ≥50 years)	1.016	0.639–1.618	0.945
pT stage (≤2 cm vs. >2 cm)	1.018	0.657–1.577	0.936
Histological grade (G I and II vs. G III)	3.164	1.965–5.094	<0.001
ER status (− vs. +)	0.776	0.398–1.515	0.458
PR status (− vs. +)	1.447	0.742–2.820	0.278
HER-2 status (− vs. +)	1.256	0.714–2.213	0.429

CTLs cytotoxic T lymphocytes; ER, estrogen receptor; PR progesterone receptor; HER-2 human epidermal growth factor receptor 2; HR hazard ratio; 95%-CI 95%-confidence interval.

a The total number of available cases for Ki-67 expression in univariate analysis is 320.

Among 285 patients with positive CD8^+^ CTLs infiltrate status, the median duration of OS was 12.98 years compared with 8.92 years among 49 patients with negative CD8^+^ CTLs infiltrate status. Patients with positive CD8^+^ CTLs infiltrate status had good survival probabilities with five and ten-year OS rate of 88.07% and 69.82%, respectively. In comparison, patients with negative CD8^+^ CTLs infiltrate status had a decrease in survival probabilities with five and ten-year OS rate of 70.21% and 44.68%, respectively. In univariate analysis, patients with positive CD8^+^ CTLs infiltrate status had longer OS than patients with negative CD8^+^ CTLs infiltrate status (P = 0.014, HR = 0.430, 95% CI = 0.220–0.840; Table 3A). Furthermore, Kaplan Meier survival analysis also visualized a significantly different OS time between patients with positive and negative CD8^+^ CTLs infiltrate status (Log-rank test: P = 0.011, Fig. 2B). Applying multivariate Cox analysis including age at diagnosis, pT stage, histological grade, ER status, PR status and HER-2 status, positive CD8^+^ CTLs infiltrate status showed an independent association with improved OS with P = 0.007 (HR = 0.387, 95% CI = 0.194–0.770; Table 3B). Moreover, Histological grade and PR status also conferred independently good prognosis values in this multivariate Cox regression model with P<0.001, HR = 3.086, 95% CI = 1.867–5.100 and P = 0.013, HR = 3.536, 95% CI = 1.300–9.616 respectively (Table 3B).

**Table 3:** Univariate analysis and multivariate Cox analysis of CD8^+^ CTLs infiltrate status for overall survival (OS) in the entire cohort (n = 332).

Clinicopathological variables	HR	95%-CI	P
A. Univariate analysis			
CD8^+^ CTLs infiltrate status (− vs. +)	0.430	0.220–0.840	0.014
Age (<50 years vs. ≥50 years)	1.188	0.647–2.182	0.579
pT stage (≤2 cm vs. >2 cm)	1.708	0.996–2.932	0.052
Histological grade (G I and II vs. G III)	3.785	2.205–6.497	<0.001
ER status (− vs. +)	0.577	0.330–1.009	0.054
PR status (− vs. +)	0.872	0.496–1.533	0.635
HER-2 status (− vs. +)	1.934	0.995–3.760	0.052
Ki-67 expression^a^ (Low vs. High)	2.221	1.287–3.832	0.004
B. Multivariate Cox analysis			
CD8^+^ CTLs infiltrate status (− vs. +)	0.401	0.202–0.797	0.009
Age (<50 years vs. ≥50 years)	1.343	0.720–2.505	0.354
pT stage (≤2 cm vs. >2cm)	1.286	0.726–2.279	0.388
Histological grade (G I and II vs. G III)	4.399	2.378–8.136	<0.001
ER status (− vs. +)	0.407	0.160–1.031	0.058
PR status (− vs. +)	3.536	1.300–9.616	0.013
HER-2 status (− vs. +)	1.634	0.822–3.247	0.161

CTLs cytotoxic T lymphocytes; ER estrogen receptor; PR progesterone receptor; HER-2 human epidermal growth factor receptor 2; HR hazard ratio; 95%-CI 95%-confidence interval.

a The total number of available cases for Ki-67 expression in univariate analysis is 320.

Performing bivariate Cox analysis, positive CD8^+^ CTLs infiltrate status had significant associations with longer DFS (P = 0.006, HR = 0.464, 95% CI = 0.267–0.806; Table 4A) as well as longer OS (P = 0.007, HR = 0.395, 95% CI = 0.201–0.773; Table 4B) independent of Ki-67 expression.

**Table 4:** Bivariate Cox analysis of CD8^+^ CTLs infiltrate status with Ki-67 expression for disease-free survival (DFS) (A) and overall survival (OS) (B) (n = 320).

Clinicopathological variables	HR	95%-CI	P
A. Disease free survival (DFS)			
CD8^+^ CTLs infiltrate status (− vs. +)	0.464	0.267–0.806	0.006
Ki-67 expression (Low vs. High)	1.936	1.264–2.964	0.002
B. Overall survival (OS)			
CD8^+^ CTLs infiltrate status (− vs. +)	0.395	0.201–0.773	0.007
Ki-67 expression (Low vs. High)	2.246	1.301–3.877	0.004

CTLs cytotoxic T lymphocytes; HR hazard ratio; 95%-CI 95%-confidence interval.

### Prognostic Value of CD8^+^ CTLs in Different Breast Cancer Subtypes

**Figure 3:**
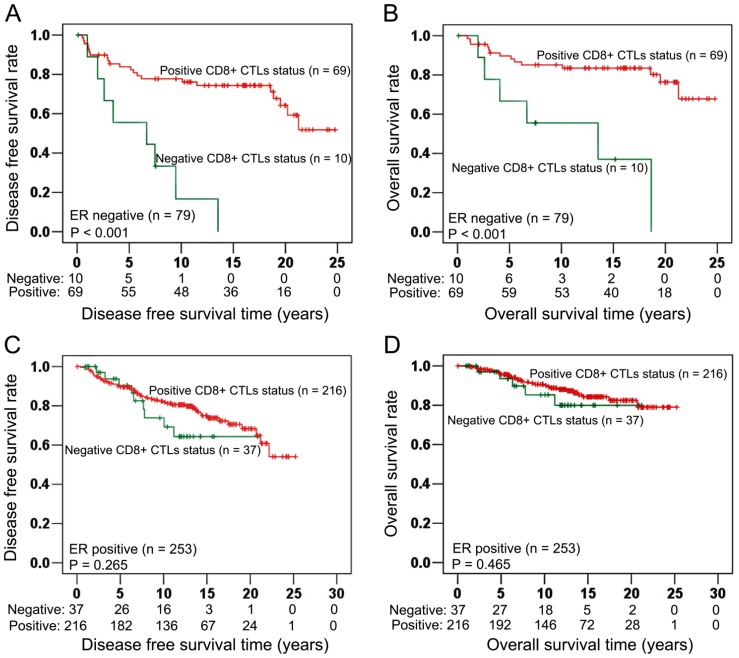
Associations of CD8^+^ CTLs with survival in ER negative (n = 79) and ER positive (n = 253) cancers. In ER negative cancers (n = 79), positive CD8^+^ CTLs infiltrate status had significant associations with longer DFS (Log-rank test: P<0.001; Fig. 3A) and longer OS (Log-rank test: P<0.001; Fig. 3B). In ER positive cancers (n = 253), Kaplan Meier survival analysis illustrated that there were no significant associations between CD8^+^ CTLs and DFS (Log-rank test: P = 0.265; Fig. 3C), OS (Log-rank test: P = 0.465; Fig. 3D).

In ER negative cancers (n = 79, table 5), positive CD8^+^ CTLs infiltrate status illustrated significant and strong protection effect on DFS (Log-Rank test: P<0.001, Fig. 3A). Similarly as for DFS, positive CD8^+^ CTLs infiltrate status also significantly associated with longer OS (Log-rank test: P<0.001, Fig. 3B). In contrast, CD8^+^ CTLs infiltrate status had no significant associations with DFS (Log-rank test: P = 0.265, Fig. 3C) and OS (Log-rank test: P = 0.465, Fig. 3D) in ER positive cancers (n = 253, table 5).

**Table 5:** Percentage of each breast cancer subtype.

Subtype	Number	%
ER negative	79	24
ER positive	253	76
HER-2 negative	291	88
HER-2 positive	41	12
ER, PR and HER-2 negative	50	15

ER estrogen receptor; PR progesterone receptor; HER-2 human epidermal growth factor receptor 2.

**Figure 4:**
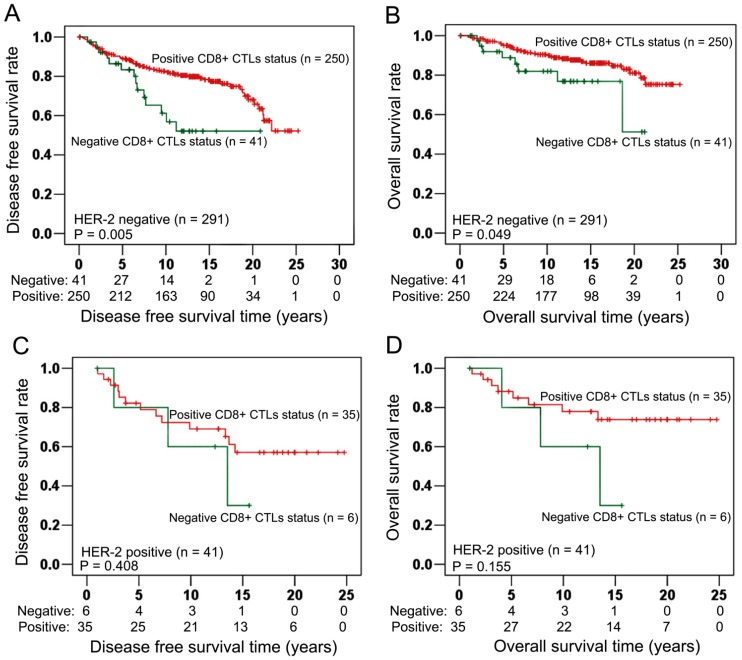
Associations of CD8^+^ CTLs with survival in HER-2 negative (n = 291) and HER-2 positive (n = 41) cancers. In HER-2 negative breast cancers (n = 291), Kaplan Meier curves showed that positive CD8^+^ CTLs infiltrate status had significantly longer DFS than negative CD8^+^ CTLs infiltrate status (Log-rank test: P = 0.005; Fig. 4A). Furthermore, longer OS was also significantly associated with positive CD8^+^ CTLs infiltrate status (Log-rank test: P = 0.049; Fig. 4B). In HER-2 positive breast cancer (n = 41), however, no significant associations were showed between CD8^+^ CTLs infiltrate status and DFS (Log-rank test: P = 0.408; Fig. 4C), OS (Log-rank test: P = 0.155; Fig. 4D).

In HER-2 negative cancer (n = 291, table 5), positive CD8^+^ CTLs infiltrate status had significant associations with improved DFS (Log-rank test: P = 0.005, Fig. 4A) and OS (Log-rank test: P = 0.049, Fig. 4B). In HER-2 positive cancer (n = 41, table 5), however, DFS (Log-rank test: P = 0.408, Fig. 4C) and OS (Log-rank test: P = 0.155, Fig. 4D) had no significant associations with CD8^+^ CTLs infiltrate status.

**Figure 5:**
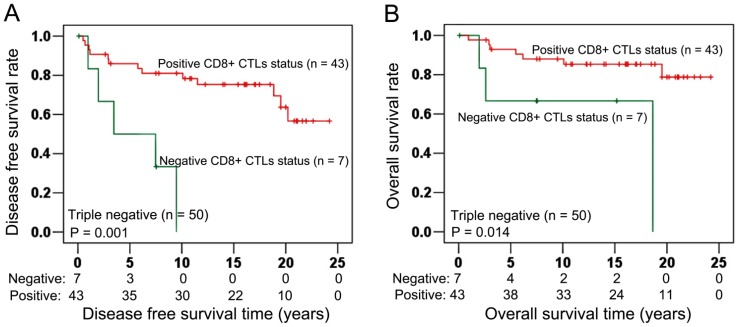
Association of CD8^+^ CTLs with survival in triple negative cancer. In ER, PR and HER-2 triple negative breast cancer, CD8^+^ CTLs infiltrate status had a strong and significant association with DFS (Log-rank test: P = 0.001; Fig. 5A). Moreover, positive CD8^+^ CTLs infiltrate status also had significant association with longer OS (Log-rank test: P = 0.014; Fig. 5B).

In ER, PR, HER-2 triple-negative cancer (n = 50, table 5), Kaplan Meier calculation visualized a significantly different DFS between positive and negative CD8^+^ CTLs infiltrate status (Log-rank test: P = 0.001, Fig. 5A). Similarly as for DFS, CD8^+^ CTLs infiltrate status also had a significant association with improved OS (Log-rank test: P = 0.014, Fig. 5B). Performing Chi-Squared tests, CD8^+^ CTLs infiltrate status showed a significant correlation with pT stage (P = 0.015). In contrast, no significant correlations were found between CD8^+^ CTLs infiltrate status and age at diagnosis (P = 0.063), histological grade (P = 0.686), ER status (P = 0.662), PR status (P = 0.843), HER-2 status (P = 0.925), Ki-67 expression (P = 0.982) (Table 6).

**Table 6:** Correlations of CD8^+^ CTLs infiltrate status with clinicopathological variables (n = 332).

Variables	CD8^+^ CTLs infiltrate status			P*
	Negative (%)	Positive (%)		
No. of patients	47 (14.2)	285 (85.8)		
Age at diagnosis				
<50	7 (2.1)	79 (23.8)		0.063
≥50	40 (12.1)	206 (62.0)		
pT stage				
≤2 cm	22 (6.6)	186 (56.1)		0.015
>2 cm	25 (7.5)	99 (29.8)		
Histological grade				
G 1 and 2	38 (11.4)	223 (67.2)		0.686
G 3	9 (2.7)	62 (18.7)		
Estrogen receptor status				
Negative	10 (3.0)	69 (20.8)		0.662
Positive	37 (11.1)	216 (65.1)		
Progesterone receptor status				
Negative	14 (4.3)		89 (26.8)	0.843
Positive	33 (9.9)		196 (59.0)	
HER-2 status				
Negative	41 (12.4)		250 (75.3)	0.925
Positive	6 (1.8)		35 (10.5)	
Ki-67 expression^a^				
Low	31 (9.7)		194 (60.6)	0.982
High	13 (4.1)		82 (24.6)	

*Correlation between variables was determined by Chi-Squared test.

CTLs cytotoxic T lymphocytes; HER-2 human epidermal growth factor receptor 2.

a The total number of available cases for Ki-67 expression is 320.

## Discussion

CD8^+^ CTLs are believed to have protective prognosis effect in many cancer types as mentioned in the introduction [15]–[24]. For breast cancer, however, the significance of CD8^+^ CTLs is still controversial [25]–[28]. By immunostaining CD8 molecule, a specific marker which comprised of an alpha chain and a beta chain covalently linked by a disulfide bond and a polypeptide in the T cell membrane, we detected the infiltration intensity of CD8^+^ CTLs in intratumoral and peritumoral compartments and then analyzed its prognostic role in 332 untreated node-negative breast cancer patients with long follow-up. Though peritumoral CD8^+^ CTLs had no protectively prognostic effect, strong infiltrate of intratumoral CD8^+^ CTLs was found to significantly associate with improved breast cancer specific OS and DFS, independent of standard prognostic and predictive characteristics such as age at diagnosis, pT stage, histological grade, ER, PR and HER-2 status as well as Ki-67 in the entire cohort of node-negative breast cancer patients in Kaplan-Meier analysis, univariate analysis and multivariate Cox analysis, and this protectively prognostic effect of CD8^+^ CTLs was especially strong in ER negative, HER-2 negative and triple-negative subtypes. Our results suggested that cellular immune response, represented by CD8^+^ CTLs, played a protective role against the development of breast cancer.

According to modern cancer immunoediting hypothesis [12]–[15], cancer progression can be divided to three phases: elimination, equilibrium and escape. In elimination and equilibrium stages, cancer cells are attacked by the dominating force such as CD8^+^ CTLs. Since some tumor cells undertake more genetic mutations and acquire some features enabling them to escape the suppression of adaptive immunity, tumour enters the last stage: the escape phase. In this stage, the tumor-inhibiting effect of CD8^+^ CTLs is suppressed by FOXP3^+^ Tregs, myeloid suppressor cells (MDSCs), neutrophils, M2 macrophages, Th2 CD4^+^ T cells and cytokines such as TGF-β, IL-6. DeNardo et al. [33] also suggest that even though CD8^+^ CTLs can destroy tumor cells in acute inflammation environment at the early initiation and growth stages, its anti-tumor effect is suppressed in the followed chronic inflammation stage if cancer cells are not completely eliminated. Above hypotheses explain why breast cancer cells still grow and metastasis despite the infiltrate of CD8^+^ CTLs.

Nevertheless, our results and other studies [25]–[28] showed that patients with positive CD8^+^ CTLs infiltrate significantly associated with improved survival of patients, implying that even though CD8^+^ CTLs were in the suppressive tumor microenvironment, they still had tumor-inhibiting effect in some extent. The study performed by Anz and colleagues [30] found that although medullary breast cancer (MBC) was strongly infiltrated by FOXP3^+^ Tregs, it still significantly associated with good survival because the number of intratumoral CD8^+^ CTLs exceeded the number of FOXP3^+^ Tregs in most MBC cases, indicating the more important role CD8^+^ CTLs played in deciding the prognosis of breast cancer. Intratumoral CD8^+^ CTLs in cancers of colorectal [15], [16], lung [17], [18], oesophageal [19], [20], epithelial ovarian [21], [22], renal cell [23] and pancreatic cancers [24] were believed to have protectively prognostic effect. We also found that only intratumoral CD8^+^ CTLs, instead of peritumoral CD8^+^ CTLs, was significantly associated with better survival of breast cancer. To our knowledge, this is the first study to have this finding in breast cancer. Our finding can be explained by a study [34] demonstrating that the proliferation of peritumoral CD8^+^ CTLs was suppressed by FOXP3^+^ Tregs which infiltrated and were then activated only in breast tumor stroma and thus associated with worse survival of patients; within tumor cells nests, in contrast, lower density FOXP3^+^ Tregs were not activated and therefore had no ability to suppress intratumoral CD8^+^ CTLs. Michael and colleagues [35] also showed that only FOXP3^+^ Tregs in stroma were activated by mature dendritic cells likely through tumor-associated antigens presentation, thus FOXP3^+^ Tregs in stroma, instead of FOXP3^+^ Tregs in tumor cells nests, were significantly associated with higher risk of relapse and death.

Our study showed that there were significant associations of CD8^+^ CTLs infiltrate status with improved prognosis of ER negative, HER-2 negative subgroups, which was supported by Mahmoud et al [25]. In addition, the strong association between positive CD8^+^ CTLs infiltrate status and better survival of triple-negative breast cancer was also found in our study, which was also consistent with the results illustrated by Liu et al [28] and Mahmoud et al [25]. Why was CD8^+^ CTLs infiltrate status only associated with better survival of hormone-independent cancers? Calabrò and colleagues [36] well reported that over-expression of immune response genes was more often identified in ER negative as compared with ER positive breast cancer. The study performed by Oh et al. [37] explained this phenomenon further. These authors found that highly proliferating breast cancer had an enhanced immune response leading to better prognosis in both ER positive and ER negative cancers. The proportions of highly proliferative cancer cells in these two subtypes, however, were different. According to their data, about 60% of ER negative cancers were highly proliferating while in ER positive cancers the proportion was only 17%. Accordingly, approximately 35% of ER positive cancers were slowly growing as compared to only 8% ER negative cancers. Interesting, about 36% of ER negative cancers had highly active immune response. The proportion of ER positive cancers with high immune response was only 20%, therefore supporting the notion that ER might have an inhibitory effect on immune response. Low proliferation activity of ER positive breast cancer might lead to an attenuated immune response and hence to a comparatively poor prognosis. In the ER negative cancers, however, a higher proportion of highly proliferative cancer cells might result in a strong immune response as reflected by a strong CD8^+^ CTLs positive infiltrate, and thus these ER negative cancers had a better survival. In HER-2 positive cancer, absolute number and percentage of circulating FOXP3^+^ Tregs were significantly and strikingly increased compared to HER-2 negative breast cancer and healthy donors. On the contrary, there was no big difference of the number and percentage of circulating FOXP3^+^ Tregs between HER-2 negative patients and healthy donors [38]. Since FOXP3^+^ Tregs suppress the proliferation and function of CD8^+^ CTLs, and its strong infiltration associate with poor prognosis, it is understandable why CD8^+^ CTLs have not protective effect on survival of HER-2 positive cancer and only associate with better prognosis of HER-2 negative breast cancer.

A potential weakness of our study is the rather small sample size of only 332 patients which might affect subgroup analysis due to variable statistical power between subgroups of differing size with varying numbers of events. A second shortcoming is the lack of an independent validation cohort of node-negative patients not treated in an adjuvant setting. A potential strength, though, is that this population allows for assessing the pure prognosis effect of a biomarker without potential predictive interaction.

In conclusion, our results illustrate that intratumoral CD8^+^ CTLs have protective effect on survival in the whole cohort and biological subtypes with ER negative, HER-2 negative and triple-negative. This shows that cellular immunity, represented by CD8^+^ CTLs, has anti-tumor activity, and may be used as immunotherapeutic tool to improve the prognosis of breast cancer patients, especially prognosis-poor biological subtypes.

### Ethical Standards

The experiments comply with the current laws of China.

## Supporting Information

Figure S1 **Associations of intratumoral CD8^+^ CTLs positive infiltrate score 0 vs. 1+, 0 vs. 2+ and 0 vs. 3+ with survival.** Performed Log-Rank tests and Kaplan Meier estimates, there were significant associations of intratumoral CD8^+^ CTLs score 0 vs. 1+ with DFS (Log-rank test: P = 0.013, Figure S1A) and OS (Log-Rank test: P = 0.033, Figure S1B); Intratumoral CD8^+^ CTLs score 0 vs. 2+ also significantly associated with DFS (Log-Rank test: P = 0.006, Figure S1C) and had a trend correlation with OS (Log-rank test: P = 0.056, Figure S1D); Similarly, DFS (Log-rank test: P = 0.019, Figure S1E) and OS (Log-Rank test: P = 0.012, Figure S1F) were significantly associated with intratumoral CD8^+^ CTLs score 0 vs. 3+.
(TIF)

Figure S2 **Associations of intratumoral CD8^+^ CTLs positive infiltrate score 1+ vs. 2+, 1+ vs. 3+ and 2+ vs. 3+ with prognosis.** Performed Log-Rank tests and Kaplan Meier estimates, there were no survival differences between intratumoral CD8^+^ CTLs infiltrate score 1+ and 2+ (Log-rank test: P = 0.161 for DFS, Figure S2A; P = 0.727 for OS, Figure S2B), 1+ and 3+ (Log-rank test: P = 0.814 for DFS, Figure S2C; P = 0.421 for OS, Figure S2D), 2+ and 3+ (Log-rank test: P = 0.388 for DFS, Figure S2E; P = 0.547 for OS, Figure S2F).
(TIF)

Figure S3 **Associations of peritumoral CD8^+^ CTLs positive infiltrate score 0 vs. 1+, 0 vs. 2+ and 0 vs. 3+ with survival.** Survival of breast cancer were not significantly associated with peritumoral CD8^+^ CTLs positive infiltrate score 0 vs. 1+ (Log-rank test: P = 0.104 for DFS, Figure S3A; P = 0.227 for OS, Figure S3B), peritumoral CD8^+^ CTLs positive infiltrate score 0 vs. 2+ (Log-rank test: P = 0.070 for DFS, Figure S3C; P = 0.186 for OS, Figure S3D), peritumoral CD8^+^ CTLs positive infiltrate score 0 vs. 3+ (Log-rank test: P = 0.140 for DFS, Figure S3E; P = 0.084 for OS, Figure S3F).
(TIF)

Figure S4 **Associations of peritumoral CD8^+^ CTLs positive infiltrate score 1+ vs. 2+, 1+ vs. 3+ and 2+ vs. 3+ with survival.** There were no significant associations of survival with peritumoral CD8^+^ CTLs positive infiltrate score 1+ vs. 2+ (Log-rank test: P = 0.905 for DFS, Figure S4A; P = 0.950 for OS, Supple Figure S4B), 1+ vs. 3+ (Log-rank test: P = 0.811 for DFS, Figure S4C; P = 0.355 for OS, Figure S4D), 2+ vs. 3+ (Log-rank test: P = 0.885 for DFS, Figure S4E; P = 0.423 for OS, Figure S4F).
(TIF)

Figure S5 **Associations of intratumoral CD8^+^ CTLs positive infiltrate scores with prognosis.** Intratumoral CD8^+^ CTLs positive infiltrate score 0, 1+, 2+ and 3+ had significant correlation with DFS (Log-rank test: P = 0.014, Figure S5A) and a trend correlation with OS (Log-rank test: P = 0.070, Figure S5B); However, there were no significant associations of intratumoral CD8^+^ CTLs positive infiltrate score 1+, 2+, 3+ with DFS (Log-rank test: P = 0.358, Figure S5C) and OS (Log-rank test: P = 0.709, Figure S5D).
(TIF)

Figure S6 **Associations of peritumoral CD8^+^ CTLs positive infiltrate scores with prognosis.** No significant associations were found between prognosis and peritumoral CD8^+^ CTLs positive infiltrate score 0, 1+, 2+, 3+ (Log-rank test: P = 0.368 for DFS, Figure S6A; P = 0.392 for OS, Figure S6B), prognosis and peritumoral CD8^+^ CTLs positive infiltrate score 1+, 2+, 3+ (Log-rank test: P = 0.967 for DFS, Figure S6C; P = 0.638 for OS, Figure S6D).
(TIF)
