# Supplementary figures and images for: Intratumoral CD8+ Cytotoxic Lymphocyte Is a Favorable Prognostic Marker in Node-Negative Breast Cancer
Source: PLoS One. 2014 Apr 17;9(4):e95475. doi: 10.1371/journal.pone.0095475 (PMC3990637; doi:10.1371/journal.pone.0095475)

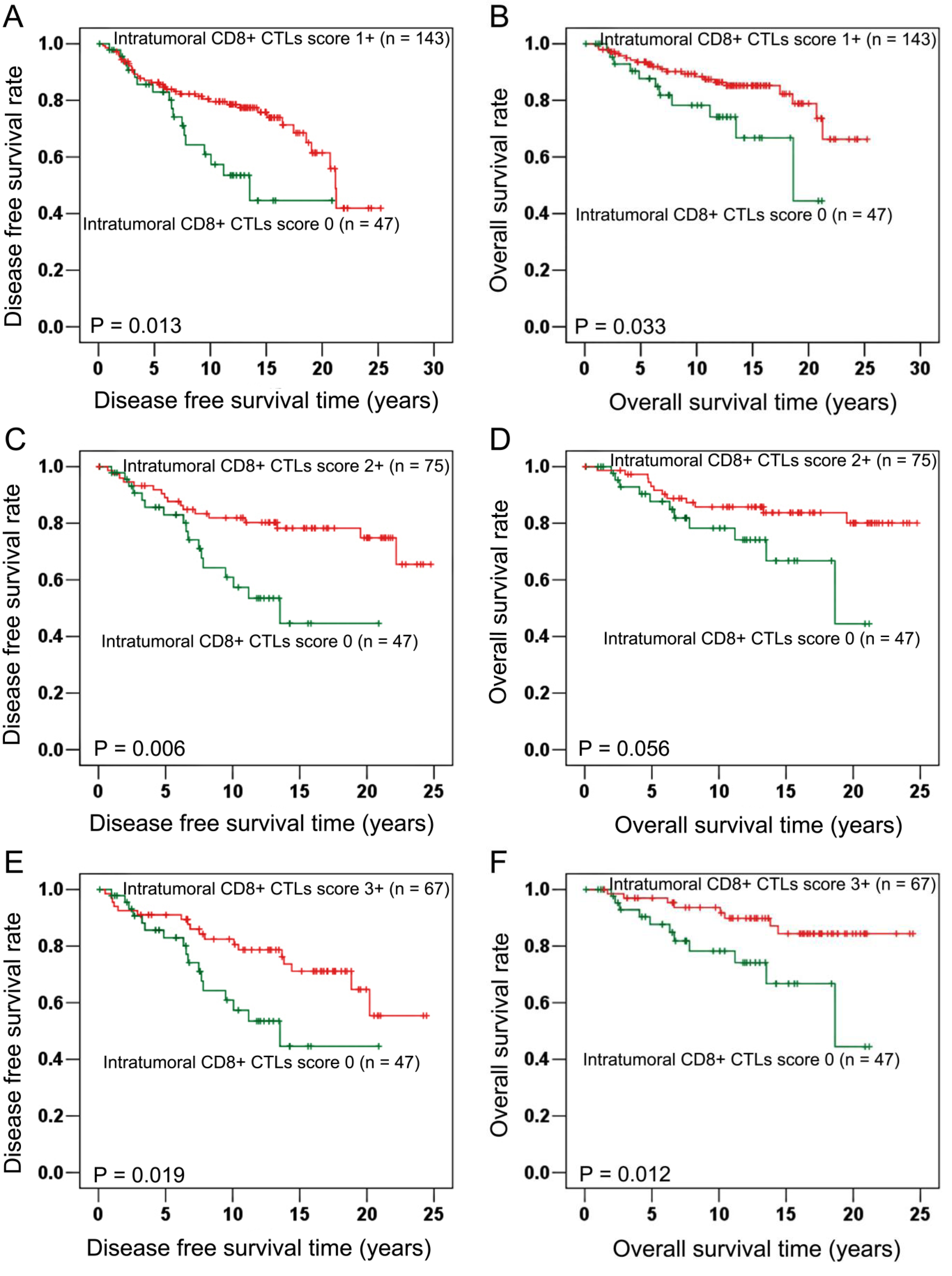

Supplement: Figure S1 — Associations of intratumoral CD8+ CTLs positive infiltrate score 0 vs. 1+, 0 vs. 2+ and 0 vs. 3+ with survival. Performed Log-Rank tests and Kaplan Meier estimates, there were significant associations of intratumoral CD8+ CTLs score 0 vs. 1+ with DFS (Log-rank test: P = 0.013, Figure S1A) and OS (Log-Rank test: P = 0.033, Figure S1B); Intratumoral CD8+ CTLs score 0 vs. 2+ also significantly associated with DFS (Log-Rank test: P = 0.006, Figure S1C) and had a trend correlation with OS (Log-rank test: P = 0.056, Figure S1D); Similarly, DFS (Log-rank test: P = 0.019, Figure S1E) and OS (Log-Rank test: P = 0.012, Figure S1F) were significantly associated with intratumoral CD8+ CTLs score 0 vs. 3+. (TIF) [file pone.0095475.s001.tif]

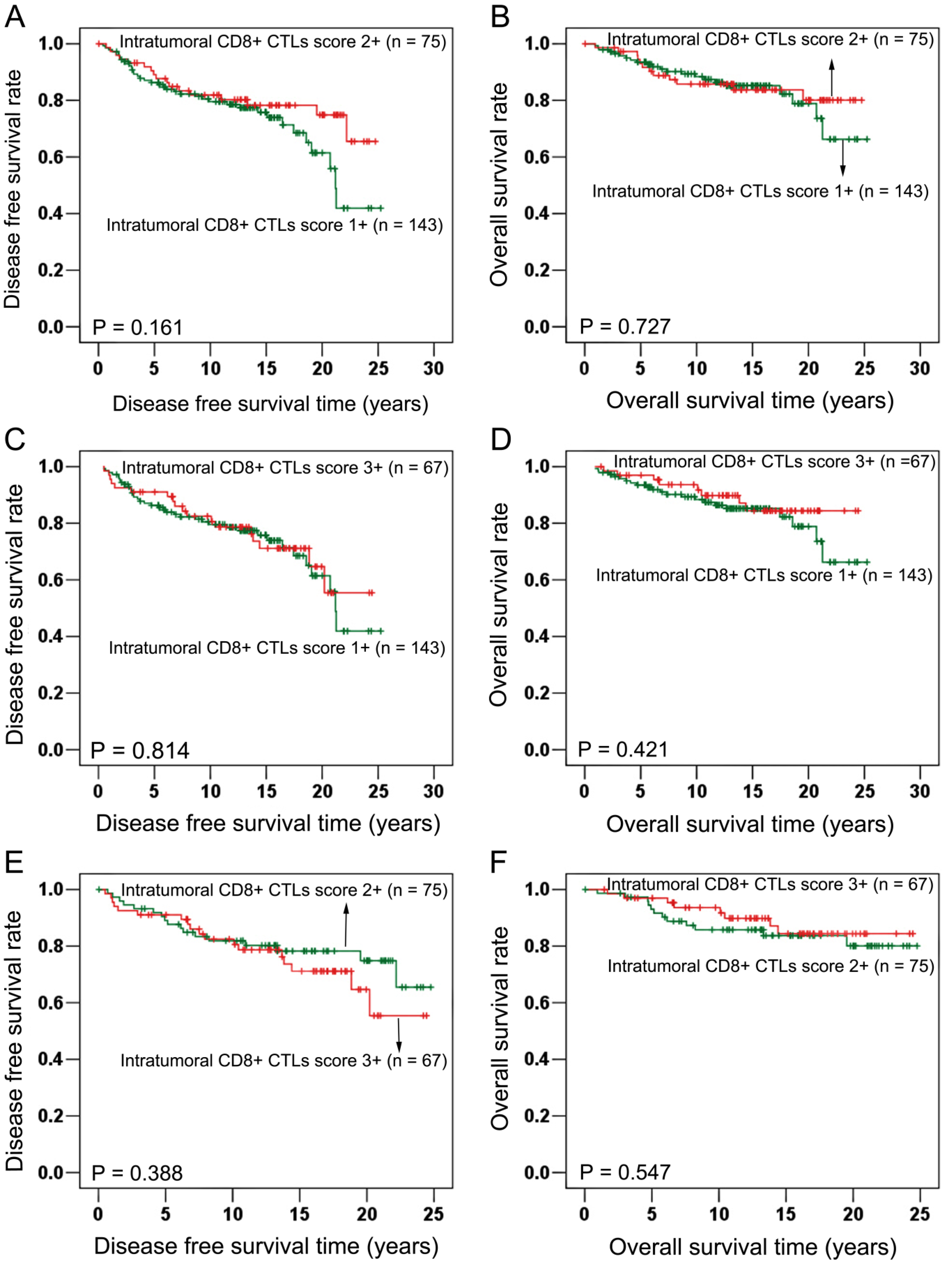

Supplement: Figure S2 — Associations of intratumoral CD8+ CTLs positive infiltrate score 1+ vs. 2+, 1+ vs. 3+ and 2+ vs. 3+ with prognosis. Performed Log-Rank tests and Kaplan Meier estimates, there were no survival differences between intratumoral CD8+ CTLs infiltrate score 1+ and 2+ (Log-rank test: P = 0.161 for DFS, Figure S2A; P = 0.727 for OS, Figure S2B), 1+ and 3+ (Log-rank test: P = 0.814 for DFS, Figure S2C; P = 0.421 for OS, Figure S2D), 2+ and 3+ (Log-rank test: P = 0.388 for DFS, Figure S2E; P = 0.547 for OS, Figure S2F). (TIF) [file pone.0095475.s002.tif]

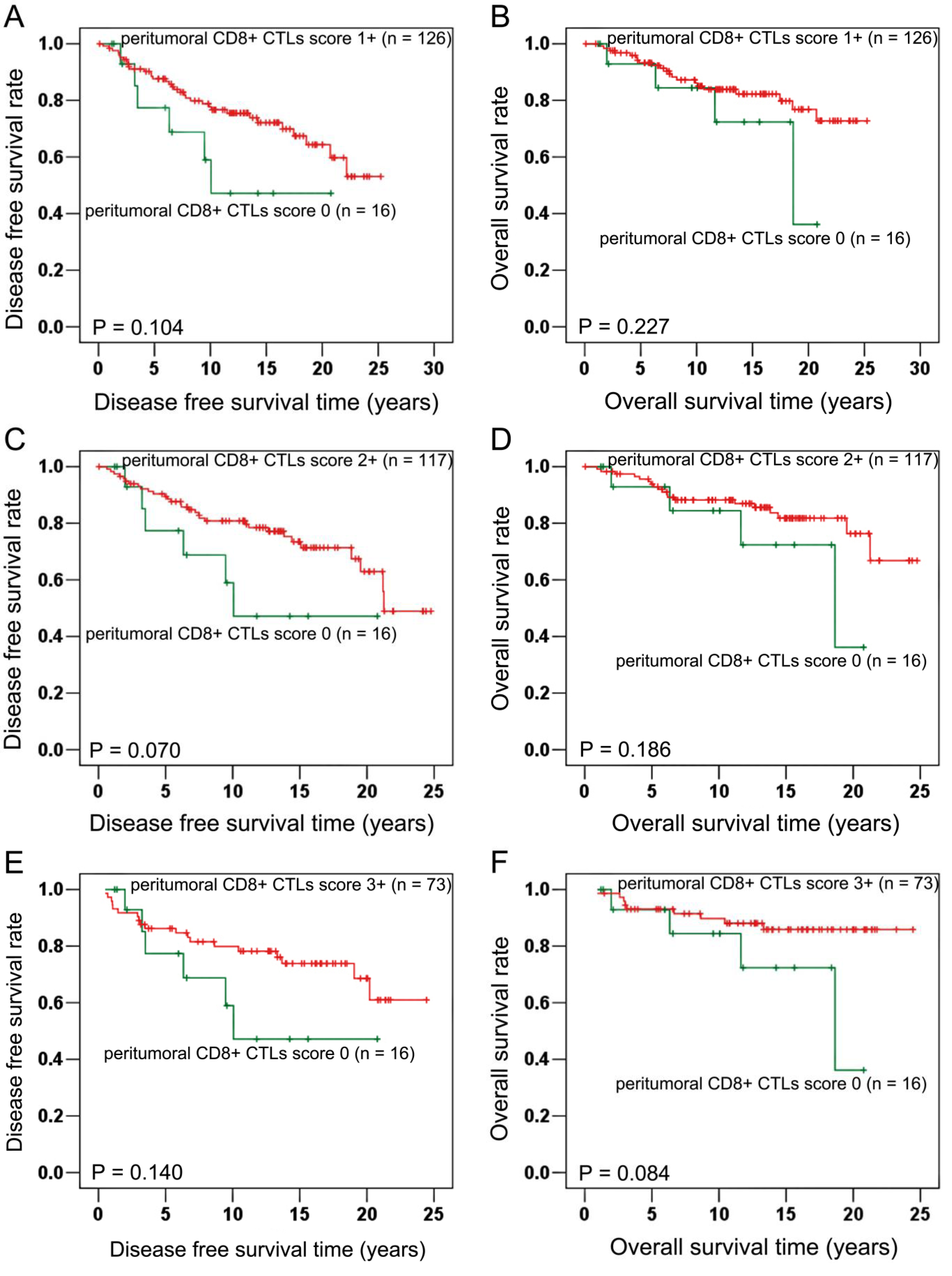

Supplement: Figure S3 — Associations of peritumoral CD8+ CTLs positive infiltrate score 0 vs. 1+, 0 vs. 2+ and 0 vs. 3+ with survival. Survival of breast cancer were not significantly associated with peritumoral CD8+ CTLs positive infiltrate score 0 vs. 1+ (Log-rank test: P = 0.104 for DFS, Figure S3A; P = 0.227 for OS, Figure S3B), peritumoral CD8+ CTLs positive infiltrate score 0 vs. 2+ (Log-rank test: P = 0.070 for DFS, Figure S3C; P = 0.186 for OS, Figure S3D), peritumoral CD8+ CTLs positive infiltrate score 0 vs. 3+ (Log-rank test: P = 0.140 for DFS, Figure S3E; P = 0.084 for OS, Figure S3F). (TIF) [file pone.0095475.s003.tif]

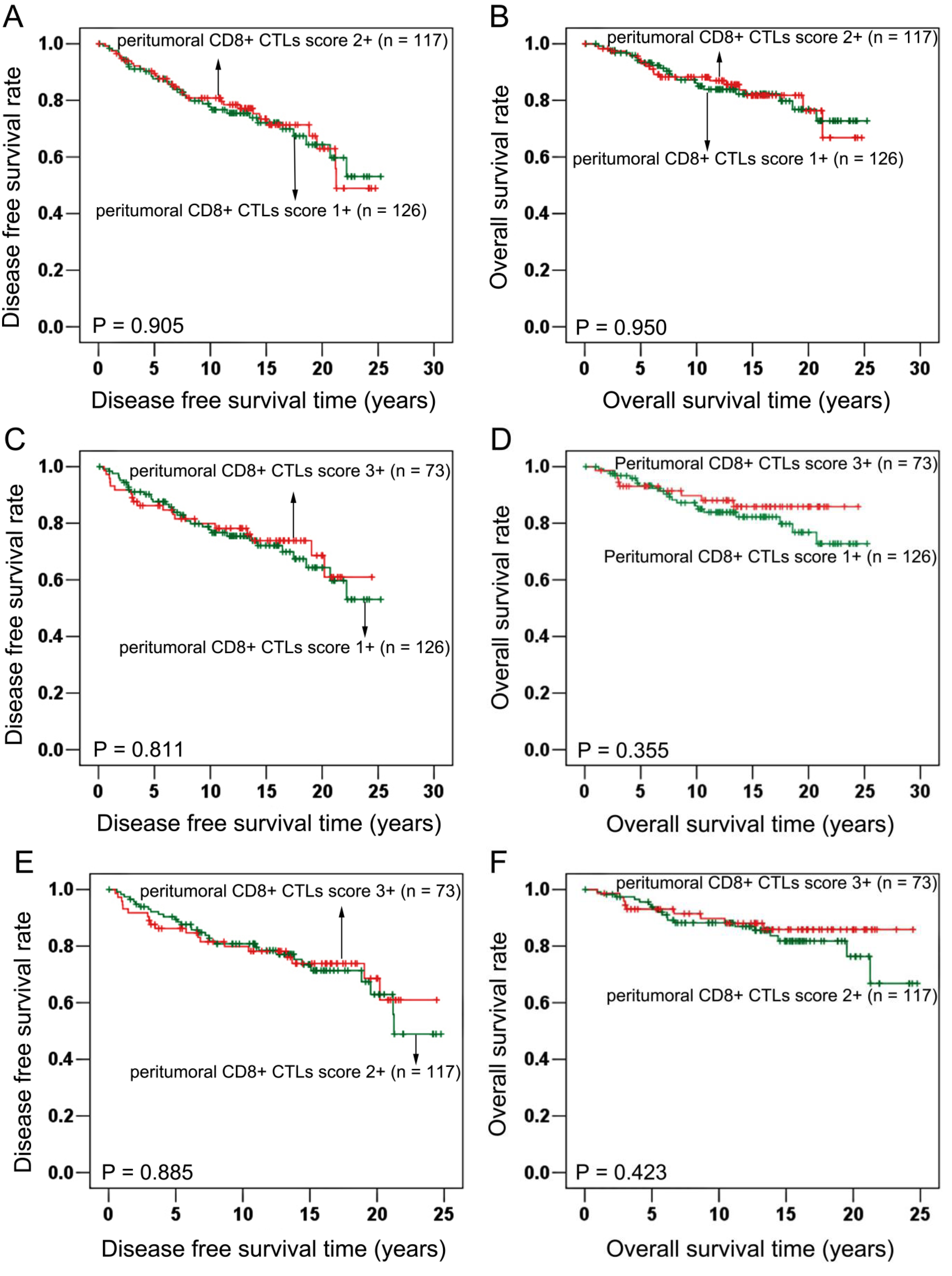

Supplement: Figure S4 — Associations of peritumoral CD8+ CTLs positive infiltrate score 1+ vs. 2+, 1+ vs. 3+ and 2+ vs. 3+ with survival. There were no significant associations of survival with peritumoral CD8+ CTLs positive infiltrate score 1+ vs. 2+ (Log-rank test: P = 0.905 for DFS, Figure S4A; P = 0.950 for OS, Supple Figure S4B), 1+ vs. 3+ (Log-rank test: P = 0.811 for DFS, Figure S4C; P = 0.355 for OS, Figure S4D), 2+ vs. 3+ (Log-rank test: P = 0.885 for DFS, Figure S4E; P = 0.423 for OS, Figure S4F). (TIF) [file pone.0095475.s004.tif]

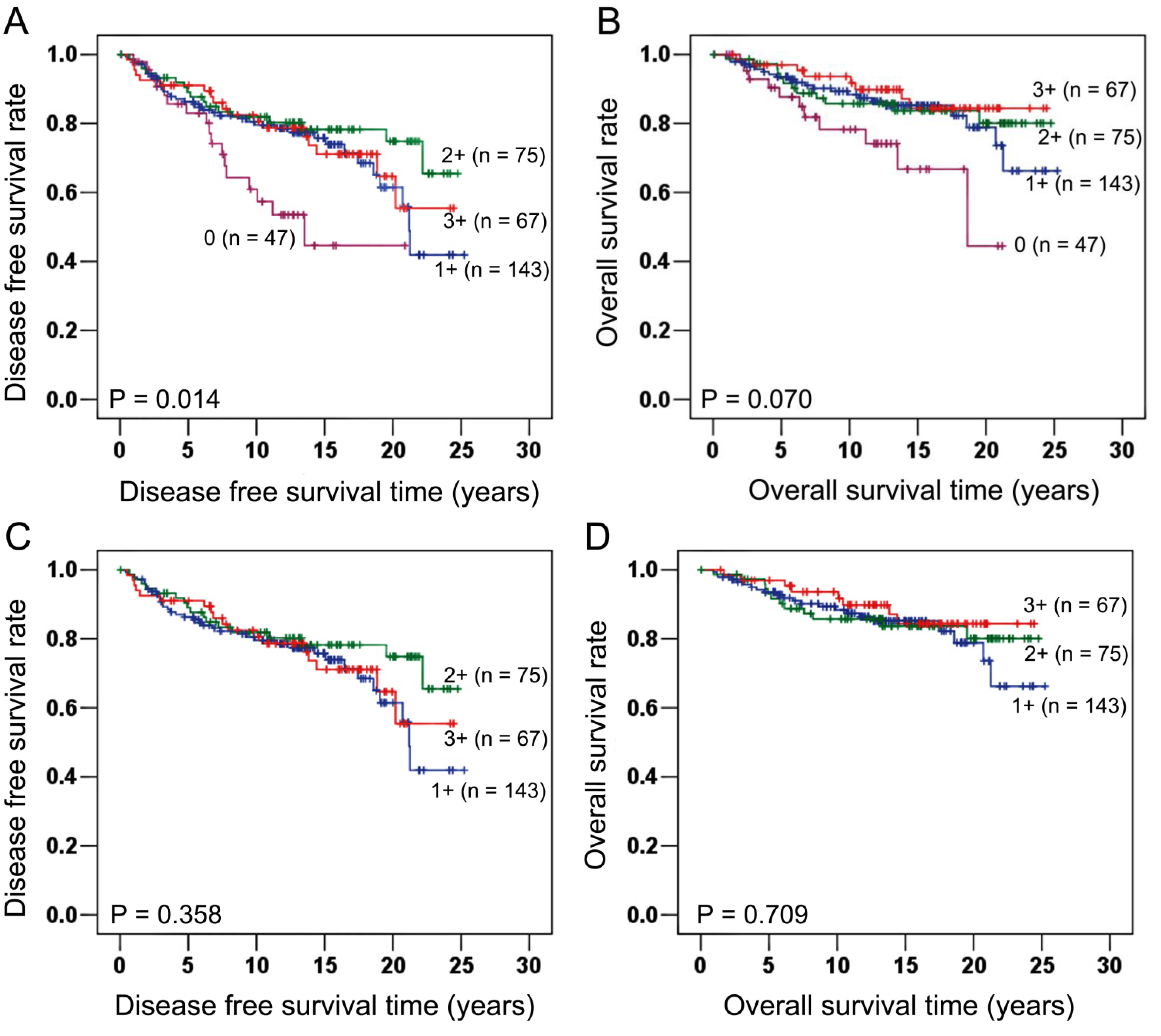

Supplement: Figure S5 — Associations of intratumoral CD8+ CTLs positive infiltrate scores with prognosis. Intratumoral CD8+ CTLs positive infiltrate score 0, 1+, 2+ and 3+ had significant correlation with DFS (Log-rank test: P = 0.014, Figure S5A) and a trend correlation with OS (Log-rank test: P = 0.070, Figure S5B); However, there were no significant associations of intratumoral CD8+ CTLs positive infiltrate score 1+, 2+, 3+ with DFS (Log-rank test: P = 0.358, Figure S5C) and OS (Log-rank test: P = 0.709, Figure S5D). (TIF) [file pone.0095475.s005.tif]

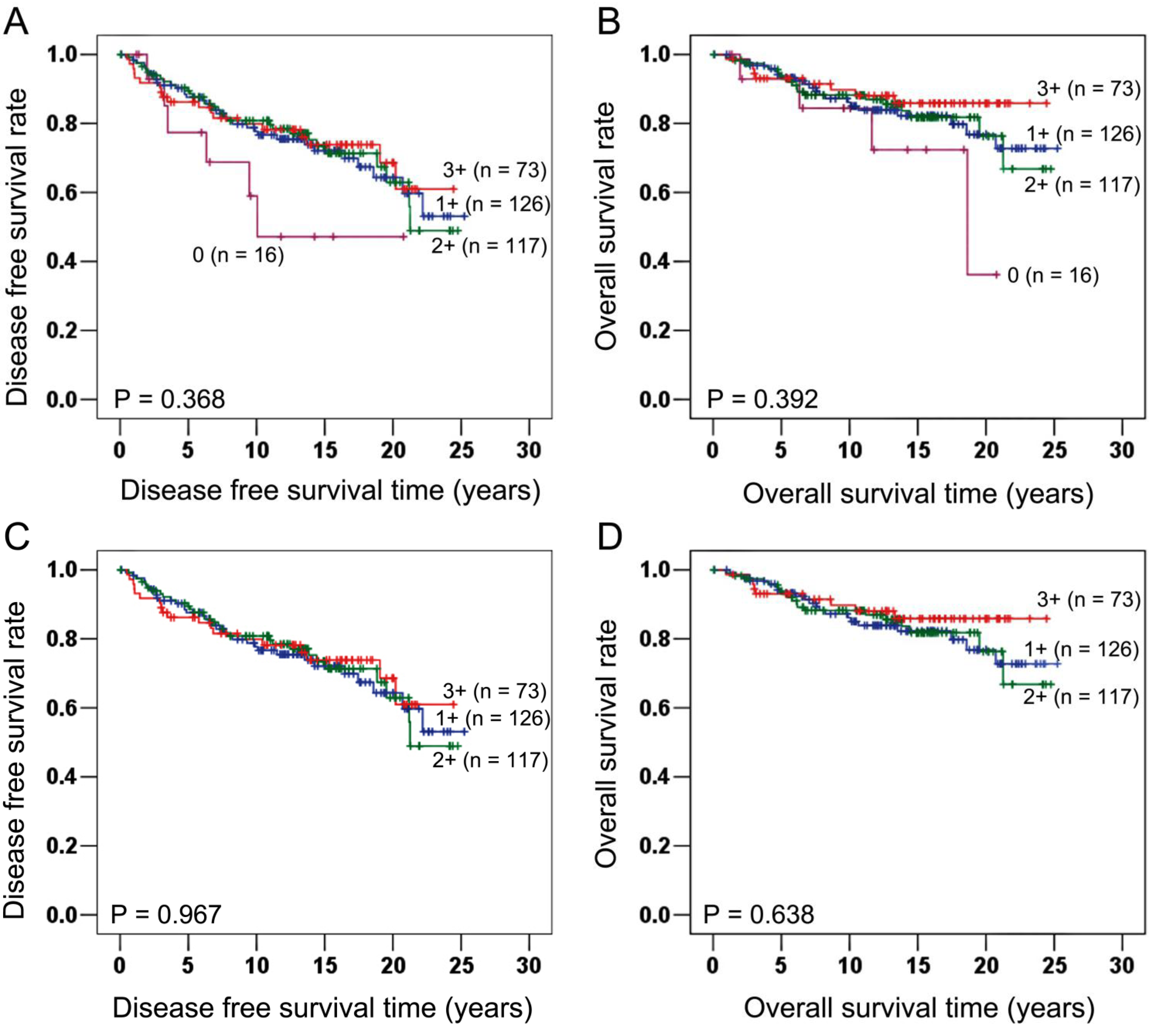

Supplement: Figure S6 — Associations of peritumoral CD8+ CTLs positive infiltrate scores with prognosis. No significant associations were found between prognosis and peritumoral CD8+ CTLs positive infiltrate score 0, 1+, 2+, 3+ (Log-rank test: P = 0.368 for DFS, Figure S6A; P = 0.392 for OS, Figure S6B), prognosis and peritumoral CD8+ CTLs positive infiltrate score 1+, 2+, 3+ (Log-rank test: P = 0.967 for DFS, Figure S6C; P = 0.638 for OS, Figure S6D). (TIF) [file pone.0095475.s006.tif]
